# Kaixin San Jiawei granule improves cognitive function and alleviates neuronal damage in Alzheimer’s disease via multi-component and multi-target mechanisms

**DOI:** 10.3389/fphar.2025.1650534

**Published:** 2025-09-19

**Authors:** Wei Liu, Yanan Zhao, Tingting Liu, Yilei Wang, Dongli Yin, Shengcan Zou, Chunze Zou, Zunlu Zhang, Hongwei Zhi, Yahan Wang

**Affiliations:** ^1^ Department of Neurology, Hunan Provincial Hospital of Integrated Traditional Chinese and Western Medicine, Changsha, China; ^2^ First College of Clinical Medicine, Shandong University of Traditional Chinese Medicine, Jinan, China; ^3^ Department of Neurology, Shandong University of Traditional Chinese Medicine Affiliated Hospital, Jinan, China; ^4^ Qingdao Chenland Pharmaceutical Co., Ltd., Qingdao, China; ^5^ University of California, Irvine, Irvine, CA, United States

**Keywords:** Kaixin San Jiawei granule, Alzheimer’s disease, network pharmacology, cognitive function, apoptosis, inflammatory cytokines, acetylcholine, therapy

## Abstract

**Background:**

Kaixin San Jiawei Granule (KSG) is a traditional Chinese medicine formulation derived from classical prescriptions. Although it has shown promise in treating Alzheimer’s disease (AD), its precise mechanisms of action remain unclear. This study aimed to systematically investigate the molecular mechanisms underlying KSG’s therapeutic effects on AD through an integrative approach combining network pharmacology with experimental validation.

**Methods:**

An *in vivo* AD model was established in male KM mice via intraperitoneal injection of scopolamine. Cognitive function was assessed using the Morris water maze, and hippocampal levels of acetylcholine (ACh), acetylcholinesterase (AChE), glutathione peroxidase (GSH-Px), and reactive oxygen species (ROS) were measured using ELISA. *In vitro*, PC12 cells were exposed to Aβ_25-35_ to induce apoptosis. Immunofluorescence staining, Western blotting, and qPCR were used to assess the expression of amyloid-beta (Aβ), apoptosis-related protein caspase-3, and inflammatory cytokines (TNF-α, IL-1β). Active components of KSG and their potential targets and pathways were identified using mass spectrometry and network pharmacology, while partial validation was performed using molecular docking and Western blotting.

**Results:**

*In vivo*, KSG significantly alleviated scopolamine-induced cognitive deficits in mice. Treatment increased hippocampal levels of ACh and GSH-Px while reducing AChE and ROS. *In vitro*, KSG mitigated Aβ_25-35_-induced cytotoxicity in PC12 cells, decreased Aβ accumulation, and downregulated the expression of TNF-α and IL-1β. However, KSG had no significant effect on telomerase activity, telomere length, or the expression of the telomere-associated protein POT1. Mass spectrometry and network pharmacology analyses identified genistein, quercetin, and apigenin as key active compounds with TP53, AKT1, PTGS2, and CNR2 identified as core targets. Molecular docking validation confirmed the favorable binding activity between them. The calcium signaling, PI3K-Akt, and MAPK pathways emerged as the primary enriched pathways.

**Conclusion:**

KSG improves cognitive function and attenuates Aβ-induced neuronal damage in AD through multi-component, multi-target synergistic mechanisms. These effects appear to be mediated by modulation of the cholinergic system, inhibition of oxidative stress and inflammation, and suppression of neuronal apoptosis. These findings provide a theoretical basis and experimental support for developing novel AD therapies based on traditional Chinese medicine.

## 1 Introduction

Alzheimer’s disease (AD) is the most common cause of dementia, accounting for 60%–80% of all cases of cognitive impairment (Marvi et al., 2025; Scheltens et al., 2021). Currently, approximately 55 million people worldwide are affected by AD, and this number is projected to rise to 152 million by 2050, representing a nearly threefold increase in global prevalence (Chen et al., 2024; Scheltens et al., 2021). AD is a chronic neurodegenerative disorder characterized by progressive cognitive decline, along with behavioral and psychological impairments (Jack et al., 2024; Twarowski and Herbet, 2023). The primary pathological features of AD include senile plaques (SPs) resulting from β-amyloid (Aβ) deposition, neurofibrillary tangles (NFTs) caused by hyperphosphorylated tau protein, and widespread neuronal loss (Jack et al., 2018). The pathogenesis of AD is complex and remains incompletely understood. Major hypothesis include Aβ accumulation, tau hyperphosphorylation, cholinergic synaptic dysfunction, oxidative stress, inflammatory damage, calcium homeostasis imbalance, and neuronal apoptosis (Jack et al., 2024). Among these, the amyloid cascade hypothesis remains the prevailing theory, making Aβ clearance a key therapeutic target (Ju and Tam, 2022). Drugs such as rokanizumab and aducanumab, which target Aβ, have been approved by the US FDA; however, their clinical application is limited by challenges such as biomarker collection, high cost, and limited efficacy (Cummings et al., 2024; Söderberg et al., 2023). Therefore, there is an urgent need to identify safer, more effective therapeutic strategies for AD.

Kaixin San Jiawei Granule (KSG) is a traditional Chinese medicine (TCM) compound derived from classical prescriptions and has demonstrated significant clinical effectiveness in enhancing cognitive function (Zhu et al., 2017). KSG is composed of multiple herbal ingredients, including ginseng, Cistanche, Polygala, Salvia miltiorrhiza, Poria cocos, Acorus tatarinowii and Rehmannia glutinosa, and is administered orally (Cao et al., 2023). Accumulating evidence supports the potential use of KSG in AD treatment. Specifically, It has been shown to upregulate neprilysin (NEP) expression in the hippocampus, promote Aβ degradation, reduce hippocampal Aβ burden, repair Aβ-induced neuronal damage, regulate amino acid and lipid metabolism, and mitigate neuronal injury, ultimately slowing cognitive decline (Wang et al., 2020; Wang et al., 2019). Additionally, KSG modulates postsynaptic AMPA receptor (AMPAR) expression to alleviate Aβ-induced neurotoxicity, and may exert neuroprotective effects by inhibiting ferroptosis, thereby improving learning and memory (Cao et al., 2023; Zhang et al., 2023). Given the central role of Aβ pathology in AD, targeting Aβ accumulation and toxicity remains an important therapeutic target.

Accordingly, this study aimed to investigate the pharmacological mechanisms of KSG in the treatment of AD using an integrated approach that combines mass spectrometry with network pharmacology. This includes exploring the mechanisms underlying drug action by examining the improvement of We further investigate KSG’s efficacy in ameliorating cognitive dysfunction in a scopolamine-induced AD mouse model and in protecting PC12 cells from Aβ-induced apoptosis. These findings are intended to provide a scientific basis for further experimental research and the rational clinical application of KSG in AD treatment.

## 2 Materials and methods

### 2.1 Animals

Ninety specific pathogen-free male KM mice (6 weeks old, body weight 20 ± 2 g) were purchased from Beijing Weitong Lihua Laboratory Animal Technology Co., Ltd. (license number: SCXK [Beijing] 20210006). The mice were housed in groups of five per cage under controlled conditions: temperature 21 °C ± 2 °C, humidity 50% ± 10%, and a 12-h light-dark cycle (lights on at 20:00, lights off at 8:00). All animal experiments were conducted in accordance with the NIH guidelines for the care and use of laboratory animals approved by the Institutional Animal Care and Use Committee (IACUC) under ethics approval number HIN-SDUTCM20231130021. Animals were adaptively fed for 1 week prior to the start of experiments.

### 2.2 Cell culture

PC-12 cells (a rat adrenal medullary pheochromocytoma cell line) were obtained from the Cell Bank of the Chinese Academy of Sciences (Shanghai, China). The cells were cultured in Dulbecco’s Modified Eagle Medium (DMEM, Cytiva, United States) supplemented with 10% fetal bovine serum (FBS, Gibco, United States) and 1% penicillin/streptomycin (P/S, Cyagen, China), and maintained at 37 °C in a humidified incubator with 5% CO_2_. When cell confluence reached approximately 80%, subculturing was performed. Cells at the third passage were used for subsequent experiments.

### 2.3 Drug preparation and molding

#### 2.3.1 Animal drug preparation and administration grouping

Scopolamine (Shanghai Macklin Biochemical Technology Co., Ltd., China) was dissolved in saline (Biosharp, China) at a concentration of 0.2 mg/mL, with an injection volume calculated as 0.1 mL per 10 g of mouse body weight. KSG (Qingdao Chenland Pharmaceutical Co., Ltd., China) was prepared at three different concentrations by dissolving 2.33 g, 4.667 g, and 9.33 g of KSG in 20 mL of double-distilled water, resulting in low-dose (KL: 116.6 mg/mL), medium-dose (KM: 233.3 mg/mL), and high-dose (KH: 466.6 mg/mL) solutions. These solutions were stored at 4 °C, and the daily gavage volume was calculated as 0.1 mL per 10 g of body weight. Donepezil (Eisai Pharmaceutical Co., Ltd., China) was weighed at 3 mg and dissolved in 20 mL of double-distilled water to prepare a 0.15 mg/mL solution, also stored at 4 °C for daily gavage administration.

After 7 days of adaptive feeding, mice were randomly divided into six groups (15 mice per group): (1) Control group (Con): saline injection + saline gavage; (2) Model group (AD): scopolamine injection + saline gavage; (3) Donepezil group (DNZ): scopolamine injection + donepezil gavage; (4) Low-dose KSG group (KL): scopolamine injection + KL gavage; (5) Medium-dose KSG group (KM): scopolamine injection + KM gavage; (6) High-dose KSG group (KH): scopolamine injection + KH gavage. All groups received the assigned gavage treatments once daily over seven consecutive days. Behavioral tests were conducted on the day after the final treatment. On the test day, gavage treatments intraperitoneal (i.p.) injections (saline or scopolamine) were administered 60 and 15–20 min prior to behavioral testing, respectively (Xu et al., 2019).

#### 2.3.2 Cell drug preparation and administration grouping

Aβ_25-35_ dry powder (Sigma-Aldrich, United States, MW = 1060.27 g/mol) was dissolved in sterile physiological saline at a concentration of 471.58 μM and incubated at 37 °C for 7 days to induce fibrillation and aggregation. The solution was then stored at −20 °C for subsequent use. Before use, the Aβ solution was diluted to the desired concentration using DMEM. KSG (12.5 mg) and donepezil (5 mg) were sterilized under UV light for 30 min and subsequently dissolved in 10 mL of DMEM to prepare 1.25 mg/mL KSG and 0.5 mg/mL donepezil solutions, respectively.

PC12 cells were grouped as follows: (1) Control group (Con): PC12 cells cultured under normal conditions; (2) AD group: PC12 cells treated with Aβ; (3) Donepezil group: PC12 cells treated with Aβ + donepezil; (4) KSG group: PC12 cells treated with Aβ + KSG.

### 2.4 Novel object recognition experiment

The novel object recognition test (NOR), based on the natural tendency of mice to explore new objects, was used to evaluate short-term memory. The procedure was as follows:

Adaptation Period: On Day 1, mice were placed in the test room for 1 h to acclimate, followed by 20 min in the open field box for further familiarization with the experimental setup (Shanghai Softmaze Information Technology Co., Ltd., China). The field was cleaned with 75% ethanol between trials to eliminate odor cues.

Familiarization Period: On Day 2, following another 1-h adaptation, two identical yellow cylindrical bottles were placed om either side of the box. Each mouse was placed with its back to the objects, and exploration behavior was recorded for 5 min. Exploration was defined as probing within 2 cm using the nose or mouth; lying or walking around was excluded. The box was cleaned with 75% ethanol after each trial.

Test Period: One yellow bottle was replaced with a green conical bottle of the same size. Each mouse was placed in the box as before, and exploration times for the novel (Tn) and familiar (Tf) objects were recorded. The recognition index (RI), reflecting the learning and memory ability of mice to explore new objects, was calculated as RI = (Tn - Tf)/(Tn + Tf). Higher RI values indicated stronger short-term memory. After each trial, the box was cleaned with 30% ethanol.

### 2.5 Platform jumping experiment

The day after the NOR test, the platform jumping experiment was performed. Mice were acclimated on the platform (Jinan Yiyan Technology Development Co., Ltd., China) for 3 minutes and trained under 36 V electric stimulation for 300 s. After 24 h, the test was conducted. Mice were placed on the platform, and latency to first jump and the number of errors (jumping off) within 300 s were recorded. If no jump occurred, latency was set at 300 s and error count at 0.

### 2.6 Morris water maze test

The Morris Water Maze Test (MWM) was performed the day after the platform jumping experiment, following established protocols, over five consecutive days, divided into a positioning navigation phase and a spatial exploration phase (Shanghai Softmaze Information Technology Co., Ltd., China) (Qin et al., 2021).

Positioning Navigation Phase (Days 1–4): Melanin powder was added to the pool to obscure the platform (located in the NW quadrant, 0.8 cm below the water surface). Water depth was 21 cm, temperature 22 °C–24 °C. Each mouse received four trials daily from semi-random start points (facing the pool wall), with 15–20 min intervals. Time to find the platform (≤60 s) was recorded (success criterion: staying on the platform for ≥2 s). If not found, latency was recorded as 60 s. After reaching the platform, mice remained on it for 10 s. Average daily latency was calculated across all trials to assess spatial learning.

Spatial Exploration Phase (Day 5): The platform was removed, and mice were placed in the SE quadrant (facing the pool wall). Metrics recorded included the number of crossings at the original platform location, swimming trajectory, quadrant ratio, and time spent in the target quadrant (within 60 s), to evaluate memory retention.

### 2.7 Material acquisition

Following euthanasia, mouse hippocampi were rapidly dissected on ice, transferred into Eppendorf tubes, and stored at −180 °C for subsequent analysis.

### 2.8 Enzyme linked immunosorbent assay (ELISA)

According to kit instructions, levels of acetylcholinesterase (AchE, Cusabio Biotech Co., Ltd., China), choline acetyltransferase (ChAT, Cusabio Biotech Co., Ltd., China), acetylcholine (Ach, CLOUD-CLONE CORP., China), superoxide dismutase (SOD, Cusabio Biotech Co., Ltd., China), glutathione peroxidase (GSH-Px, Cusabio Biotech Co., Ltd., China), reactive oxygen species (ROS, DXT Testing Technology Co., Ltd., China), and malondialdehyde (MDA, Solarbio, China) in hippocampal tissue were measured. Inflammatory cytokines IL-1β (Shanghai Jianglai Biotechnology Co., Ltd., China), IL-18 (Shanghai Jianglai Biotechnology Co., Ltd., China), and TNF-α(CLOUD-CLONE CORP., China) were quantified in PC12 cell supernatants.

### 2.9 Cell counting Kit-8 (CCK-8)

Log-phase PC12 cells were harvested and adjusted to 1.0 × 104 cells/mL. A 100 µL volume was seeded per well in 96-well plates. Groups included control, multiple drug concentrations (five replicates), and a blank group (medium only). After 24 h, 10 µL of CCK-8 solution (Biosharp, China) was added per well and incubated for 1–2 h. Absorbance at 450 nm was measured using a microplate reader, and cell viability was calculated to determine the optimal drug concentration. Similarly, cells were treated with various concentrations of Aβ_25-35_ (0, 5.89, 11.79, 23.58, 37.73, 47.158 μmol/L) for 24, 48, and 72 h to determine optimal damage conditions. The protective effects of KSG and donepezil under optimal Aβ-induced damage were then assessed and expressed as inhibition rates.

### 2.10 Western blotting

Proteins were extracted using radioimmunoprecipitation assay (RIPA) buffer (Solarbio, China), and concentrations determined by BCA protein quantification kit (Vazyme, China). SDS-PAGE gels (Shanghai Yamay Biomedical Technology Co., Ltd., China) were selected according to target protein size for electrophoresis separation, followed by PVDF membrane (Merck, Germany) transfer. Membranes were incubated overnight at 4 °C with primary antibodies: Caspase-3 Rabbit pAb (1:1000, BOSTER, China), Anti-BAX Antibody (1:1000, BOSTER, China), Akt Rabbit mAb (1:1000, Cell Signaling, United States), Phospho-Akt Rabbit mAb (1:2000, Cell Signaling, United States), ERK1/2 Mouse mAb (1:500, Bioss, China), and phospho-ERK1/2 Rabbit pAb (1:1000, bioss, China). The next day, membranes were incubated with secondary antibody (1:10000, Proteintech, China) for 1 hour at room temperature. Protein levels were visualized via ECL (Meilun Biotechnology, China) and quantified using ImageJ (National Institutes of Health, United States), with β-actin (1:10000, Proteintech, China) and GADPH (1:10000, Proteintech, China) as the internal control. Relative gray values were statistically analyzed.

### 2.11 Immunofluorescence staining

Log-phase PC12 cells were prepared at 6.25 × 10^4^ cells/mL, seeded into 24-well plates (1 mL/well), and incubated overnight. The following day, the control group received DMEM (1 mL), the AD group received 5.89 μmol/L Aβ_25-35_ solution (1 mL), and the drug groups received Aβ different concentrations of KSG or donepezil for 48 h. Cells were washed with PBS (Servicebio, China), fixed with 4% paraformaldehyde (Solarbio, China), permeabilized with 0.5% Triton X-100 (Solarbio, China), and blocked with 5% goat serum (Solarbio, China). Primary antibody APP (1:200, Abclonal, China) was added and incubated overnight at 4 °C. The following day, FITC-conjugated secondary antibody (1:200, Abclonal, China) was applied in the dark for 1 hour, followed by DAPI nuclear staining (Solarbio, China). Images were collected via fluorescence microscopy (Olympus, Japan), and intensity quantified using ImageJ.

### 2.12 Determination of telomerase activity, telomere length, and POT1 mRNA expression in PC12 cells

PC12 cells (5 × 10^5^ cells/mL) were seeded in 6-well plates (1mL/well) and grouped identically to the immunofluorescence experiment. An additional negative control (NC) group without cells was included for calibration. After 48 h of incubation, cellular protein, DNA, and RNA samples were collected separately. Telomerase activity was measured using the TRAP real-time quantitative (Shanghai Haling Biotechnology Co., Ltd., China) method. Telomere length (expressed as 2^−ΔΔCt^) and the expression level of POT1 mRNA, a telomere protective protein, were analyzed via quantitative PCR (qPCR, Vazyme, China). The primer sequences, synthesized by Sangon Biotech (Shanghai) Co., Ltd. (China), are detailed in Table 1.

**TABLE 1:** Primer sequences.

Gene	Forward primer sequence (5′-3′)	Reverse primer sequence (5′-3′)
POT1	TGG​GTA​TTG​TAC​CCC​TCC​AA	GAT​GAA​GCA​TTC​CAA​CCA​CGG
36B4	CAG​CAA​GTG​GGA​AGG​TGT​AAT​CC	CCC​ATT​CTA​TCA​TCA​ACG​GGT​ACA​A
Telomere	GGT​TTT​TGA​GGG​TGA​GGG​TGA​GGG​TGA​GGG​TGA​GGG​T	TCC​CGA​CTA​TCC​CTA​TCC​CTA​TCC​CTA​TCC​CTA​TCC​CTA

### 2.13 Non-targeted metabolomics for detecting major components in KSG

#### 2.13.1 Extraction of KSG metabolites

KSG was accurately weighed (100 mg) and added with extraction solvent (500 μL) (methanol: water = 4:1, containing 10 μg/mL internal standard). The mixture was vortexed for 30 s, homogenized at 45 Hz for 4 minutes, and sonicated in an ice-water bath for 1 hour. After incubation at −40 °C for 1 hour, the samples were centrifuged at 12,000 rpm (13,800 x g, radius: 8.6 cm) for 15 min at 4 °C. The supernatant was carefully filtered through a 0.22 μm Millipore membrane and stored at −80 °C until further analysis.

#### 2.13.2 Liquid Chromatography-Tandem Mass Spectrometry analysis

Liquid Chromatography-Tandem Mass Spectrometry (LC-MS/MS) analysis was performed using a Vanquish UHPLC system (Thermo Fisher Scientific) with a Waters UPLC BEH C18 column (1.7 μm, 2.1 × 100 mm). The flow rate was 0.5 mL/min, and the injection volume was 5 μL. The mobile phase consisted of 0.1% formic acid in water (A) and 0.1% formic acid in acetonitrile (B). The multi-step linear gradient program was as follows:

0–11 min, 85%–25% A.

11–12 min, 25%–2% A.

12–14 min, 2% A.

14–14.1 min, 2%–85% A.

14.1–16 min, 85% A.

An Orbitrap Exploris 120 mass spectrometer coupled with Xcalibur software was used to obtain MS and MS/MS data in IDA mode. The mass range was 100–1500 m/z per acquisition cycle, with top four ions selected for MS/MS fragmentation. Instrument parameters were as follows: Sheath gas flow rate: 35 Arb; auxiliary gas flow rate: 15 Arb; ion transfer tube temperature: 350 °C; vaporizer temperature: 350 °C; full MS resolution: 60,000; MS/MS resolution: 15,000; collision energy: 16/32/48 (NCE mode); spray voltage: +5.5 kV (positive mode) or −4 kV (negative mode).

### 2.14 Network pharmacology

#### 2.14.1 Screening of active ingredients and targets

The KSG stock solution was analyzed by LC-MS/MS. Experimental results and component metabolites from the TCM prescription were obtained from the YATCM and TCMIO databases. Target prediction for these metabolites was performed using the ChEMBL and TCMIO databases. Metabolites were screened based on oral bioavailability (OB ≥ 30%) and drug-likeness (DL ≥ 0.18).

#### 2.14.2 Construction of the target protein interaction network

AD-related targets were retrieved from the Therapeutic Target Database (TTD) and DisGeNET. The intersection of disease targets and KSG metabolite targets was identified to determine potential therapeutic targets. Venn diagrams were generated using the “VennDiagram” package in R (Version 3.6.3). Protein-protein interaction (PPI) data for the intersected targets were obtained from the STRING database (*Homo sapiens*) and used to construct a PPI network to explore molecular mechanisms and identify key nodes.

#### 2.14.3 Gene ontology enrichment analysis and kyoto encyclopedia of genes and genomes pathway analysis

The Gene Ontology (GO) enrichment analysis was conducted using the GO database (geneontology.org), categorizing target proteins into Biological Process (GO_BP), Cellular Component (GO_CC), and Molecular Function (GO_MF). The Kyoto Encyclopedia of Genes and Genomes (KEGG) pathway analysis was conducted via the *Homo sapiens* module of the KEGG database to determine significantly enriched metabolic pathways. Target proteins were visualized using the KEGG mapping tools.

#### 2.14.4 Metabolite-target-GO and metabolite-target-KEGG pathway

##### 2.14.4.1 Interaction

The top 30 GO terms, top 20 KEGG pathways, intersected targets, and selected active metabolites were used to construct the “metabolite-target-GO” and “metabolite-target-KEGG pathway” interaction network using R version 3.6.3. The networks were then visually analyzed.

#### 2.14.5 Molecular docking analysis

Molecular docking enables the prediction of the potential therapeutic efficacy of TCM ingredients by evaluating the binding capacity between the key components and hub genes. The three-dimensional structures of the target proteins were retrieved from the RCSB PDB database (http://www.rcsb.org/), and the crystal structures of the principal components were acquired from PubChem. The binding affinities between the bioactive compounds and their corresponding targets was calculated using AutoDock Vina 1.2.3. Finally, the docking outcomes were visualized using PyMOL (version 2.2.0).

#### 2.14.6 Statistical methods

Statistical analysis and data visualization were performed using GraphPad Prism 9.0 (Version 9.0.2, GraphPad Software Inc., United States) and Microsoft Excel 2016 (Version 16.0.18827.20102, Microsoft Corp., United States). *In vivo* data are expressed as mean ± standard error of the mean (SEM). *In vitro* experimental data are presented as mean ± standard deviation (SD). Statistical comparisons between groups were performed using one-way analysis of variance (ANOVA) or the Kruskal–Wallis test, as appropriate. The significance level was set at p < 0.05.

## 3 Result

### 3.1 KSG improved the cognitive function of AD mice

#### 3.1.1 Experimental results of novel object recognition

**FIGURE 1:**
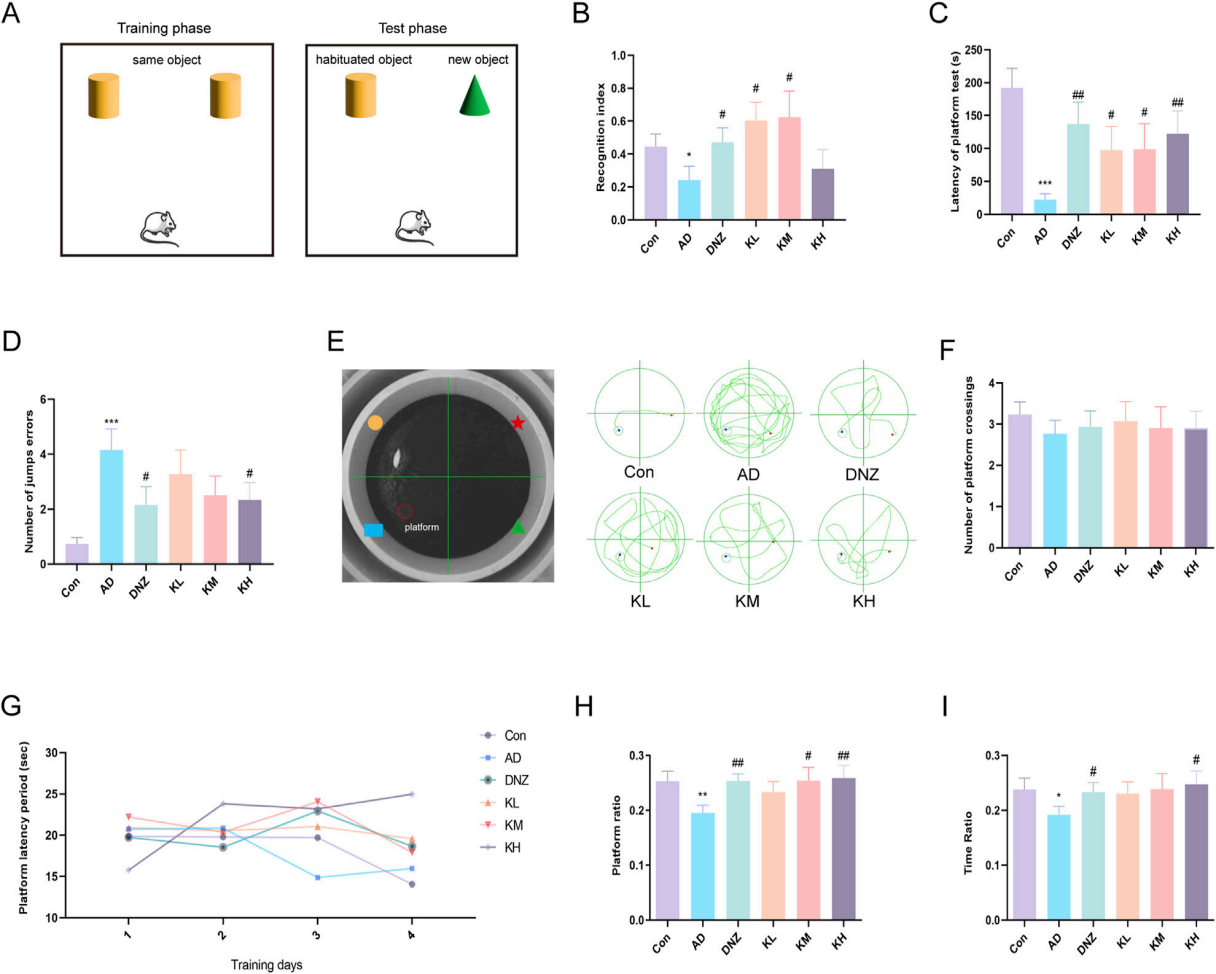
Ameliorative effects of KSG on cognitive function in AD mice. **(A)** Schematic representation of the novel object recognition test apparatus. **(B)** Statistical results of the recognition index (n = 13–15). **(C)** Latency of platform in the step-down test (n = 14–15). **(D)** Number of errors within 300 s in the step-down test (n = 14–15). **(E)** Apparatus layout of the Morris water maze and representative swimming trajectories of each group. **(F)** Number of crossings over the original platform location on day 5 of the spatial probe test (n = 14–15). **(G)** Changes in platform latency from day 1 to day 4 in the place navigation test (n = 14–15). **(H)** Percentage of swimming distance in the original platform quadrant during the spatial probe test (n = 14–15). **(I)** Percentage of swimming time in the original platform quadrant during the spatial probe test (n = 15). Data were expressed as mean ± SEM. ^*^P < 0.05, ^**^P < 0.01, and ^***^P < 0.001 vs. Con. ^#^P < 0.05, ^##^P < 0.01, and ^###^P < 0.001 vs. AD.

Mice naturally exhibit curiosity toward novel objects. Based on this behavior, the novel object recognition test (Figure 1A) was conducted to assess the effect of KSG on memory impairment in dementia model mice (Zhang, Z. et al., 2024). As shown in Figure 1B, the RI was significantly lower in AD mice compared to control mice (^*^p < 0.05), indicating that scopolamine-induced cognitive impairment caused a significant decline in short-term memory. Compared with the AD group, RI values in the donepezil, low-dose, and medium-dose KSG groups were significantly increased, with the medium-dose group exhibiting the most notable improvement. These findings suggest that KSG may reverse cognitive impairment by enhancing the preference for novel object exploration.

#### 3.1.2 Test results of jumping platform

As shown in Figures 1C,D, compared with the control group, the AD group exhibited a significant increase in the number of errors (^###^p < 0.001) and a significant reduction in jump latency (^**^p < 0.001), indicating impaired passive avoidance behavior. Compared to the AD group, the number of errors was significantly reduced in the donepezil and high-dose KSG groups (^#^p < 0.05), and jump latency was significantly increased in the donepezil, low-dose, medium-dose, and high-dose KSG groups (^#^p < 0.05). Notably, the increase in latency in the high-dose KSG group was comparable to that of the positive drug. These findings suggest that KSG may reduce cognitive errors by prolonging memory retention.

#### 3.1.3 Results of morris water maze test

The Morris water maze is commonly used to evaluate hippocampus-dependent spatial learning and memory functions (Figure 1E) (Zhang et al., 2019). In the navigation training phase, no significant differences in escape latency were observed among the groups (Figure 1G), indicating that KSG had no significant effect on learning acquisition. In the spatial probe test (Figures 1H,I), compared with the control group, the original platform quadrant run ratio and time ratio were significantly decreased in the AD group (^*^p < 0.05), suggesting impaired spatial memory. In contrast, the donepezil, medium-dose, and high-dose KSG groups showed a significant increase in the run ratio (^#^p < 0.05), while the donepezil and high-dose KSG groups exhibited a significant increase in the time ratio (^#^p < 0.05). However, no significant differences were observed among the groups in the number of platform crossings (p > 0.05) (Figure 1F). These results indicate that KSG enhances spatial memory consolidation in AD model mice, with more pronounced effects observed at medium and high doses.

### 3.2 KSG improves cholinergic system dysfunction disorder in AD mice

The cholinergic system plays a key role in maintaining brain homeostasis (Hampel et al., 2018). ACh is synthesized from choline and acetyl-CoA via ChAT and is transported into synaptic vesicles by the vesicular acetylcholine transporter (VAChT). Upon neuronal depolarization, ACh is released and binds to muscarinic or nicotinic receptors on the postsynaptic membrane to mediate signal transduction. It is subsequently hydrolyzed by AChE into choline, which is reabsorbed by the presynaptic membrane for reuse (Hampel et al., 2018; Ju and Tam, 2022). Disruption of this regulatory system is closely associated with cognitive decline in AD (Ju and Tam, 2022). Clinical studies have shown that decreased ChAT activity, combined with Aβ-induced interference in neurotransmitter synthesis, release, and metabolism, leads to reduced ACh levels, a key mechanism underlying cognitive impairment (Zhang, J. et al., 2024).

**FIGURE 2:**
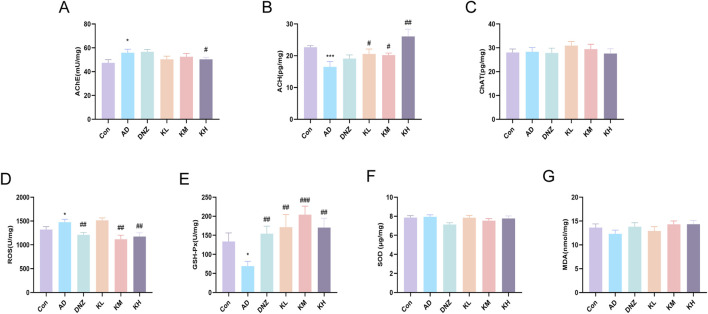
Regulatory effects of KSG on the cholinergic system and oxidative stress in AD mice. **(A)** AchE activity in the hippocampal region (n = 13–15). **(B)** ACH content in the hippocampal region (n = 14–15). **(C)** CHAT content in the hippocampal region (n = 13–15). **(D)** ROS levels in the hippocampal region (n = 14–15). **(E)** GSH-Px activity in the hippocampal region (n = 13–15). **(F)** SOD activity in the hippocampal region (n = 13–15). **(G)** MDA content in the hippocampal region (n = 14–15). Data were expressed as mean ± SEM. ^*^P < 0.05, ^**^P < 0.01, and ^***^P < 0.001 vs. Con. ^#^P < 0.05, ^##^P < 0.01, and ^###^P < 0.001 vs. AD.

In this study, ELISA results (Figures 2A–C) revealed that, compared with the Con group, hippocampal AChE levels were significantly elevated in the AD group (^*^p < 0.05), while ACh levels were markedly decreased (^**^p < 0.01, ^***^p < 0.001), confirming cholinergic dysfunction. Compared with the AD group, the high-dose KSG group significantly inhibited hippocampal AChE activity (^#^p < 0.05), and the low-, medium-, and high-dose KSG groups significantly increased ACh levels (^#^p < 0.05), exhibiting a dose-dependent trend. Notably, no significant differences in ChAT levels were observed among the groups (p > 0.05), suggesting that KSG improves cholinergic transmission efficiency primarily by inhibiting AChE activity rather than enhancing ChAT function. These findings provide direct evidence that KSG alleviates cognitive impairment by modulating ACh concentration in the synaptic cleft.

### 3.3 Regulatory effects of KSG on oxidative stress injury in AD mice

Oxidative stress results from an imbalance between reactive oxygen species (ROS) and antioxidant defenses (e.g., SOD and GSH-Px), and is closely associated with aging (Ju and Tam, 2022). AD progression is characterized by metabolic abnormalities, including oxidative stress caused by mitochondrial dysfunction (Nasb et al., 2024; Plascencia-Villa and Perry, 2021). Mitochondrial defects reduce ATP production and increase ROS, leading to membrane damage, cytoskeletal disruption, and cell death (Nasb et al., 2024). GSH-Px, a key antioxidant enzyme, removes lipid peroxides, blocks ROS-mediated damage, prevents lipid peroxidation, and protects cell membranes from oxidative injury (Reichert et al., 2020). Malondialdehyde (MDA), a lipid peroxidation byproduct, directly reflects the degree of oxidative damage (Tang et al., 2022).

ELISA results (Figures 2D,E) showed that, compared with the control group, ROS levels were significantly elevated in the AD group (*p < 0.05), while GSH-Px activity was significantly decreased (*p < 0.05), indicating reduced antioxidant capacity and intensified oxidative damage. After treatment with KSG or donepezil, ROS levels were significantly decreased in the DNZ, KM, and KH groups (##p < 0.01), and GSH-Px activity was significantly increased in the DNZ, KL, KM, and KH groups (##p < 0.01), suggesting that KSG alleviates oxidative stress by regulating both ROS production and clearance. Notably, no significant differences in SOD activity or MDA levels were observed among the groups (Figures 2F,G), which may result from compensatory SOD regulation and warrants further investigation using histopathology and dynamic metabolic profiling.

### 3.4 Neuroprotective effects of KSG in an in vitro AD model

#### 3.4.1 Effects of KSG and donepezil concentrations on PC12 cell viability

As shown in Supplementary Figure 1A, KSG concentrations ≥125 μg/mL significantly decreased cell viability compared to the control group (^*^p < 0.05). At 2,000 μg/mL, an unexpected increase in cell viability was observed, likely due to residual precipitation during mixing that was not entirely removed despite using the supernatant for measurement. Based on these findings, KSG concentrations of 15.625, 31.25, and 62.5 μg/mL were selected for subsequent experiments. As shown in Supplementary Figure 1B, donepezil concentrations ≥0.98 μg/mL also significantly reduced cell viability compared to the control group; therefore, of 0.125, 0.25, and 0.5 μg/mL were chosen for further use.

#### 3.4.2 Effects of Aβ concentrations on PC12 cell activity

Aβ induces oxidative stress in neurons, contributing to neuronal damage in AD (Calvo-Rodriguez et al., 2024). As shown in Supplementary Figure 1C–H, 48-h incubation with 5.89 μM Aβ significantly reduced PC12 cell viability compared to the control group. After 72 h of incubation, 5.89, 11.79, and 23.58 μM Aβ all significantly decreased cell viability, with the extent of reduction being too severe for further experimentation. Based on the results, 5.89 μM Aβ with 48-h incubation was selected to establish the model.

#### 3.4.3 Protective ability of KSG solution and donepezil against Aβ_25-35_-induced Apoptosis in PC12 cells

The protective abilities of KSG and donepezil are shown in the Supplementary Figure 2A–C. Overall, exposure significantly decreased PC12 cell viability in the AD group compared to the control group (^*^p < 0.05). KSG at 62.5 μg/mL further reduced viability, while all tested concentration of donepezil also showed varying degrees of reduction. However, KSG at 15.625 and 31.25 μg/mL significantly reversed the decreased Aβ-induced loss of viability (^#^p < 0.05).

Based on the concentration screening, KSG alleviated PC12 cells from Aβ-induced damage at concentrations ≤31.25 μg/mL, with the strongest effect at 15.625 μg/mL. Therefore, subsequent experiments used concentration of 7.81, 15.625, and 31.25 μg/mL. To ensure drug efficacy, this range was selected accordingly. Donepezil showed no significant protective effect on the reduced viability of PC12 cells caused by Aβ within the selected concentration range, and its concentrations for further use were set at 0.125, 0.25, and 0.5 μg/mL to avoid toxicity. These results suggest a possible threshold for the neuroprotective effect of KSG and highlight the need for careful monitoring of donepezil concentration due to its narrow safety margin.

#### 3.4.4 Effects of different drug concentrations on Aβ deposition in PC12 cells Induced by Aβ_25-35_

**FIGURE 3:**
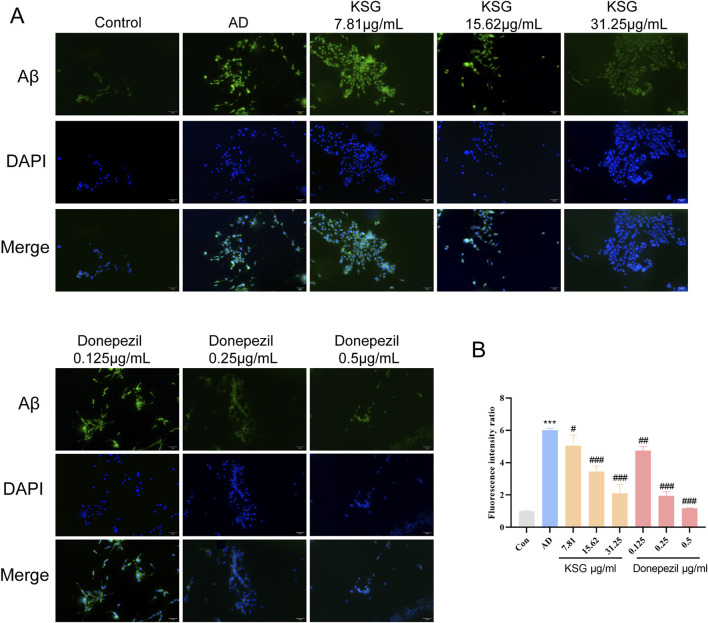
Immunofluorescence staining of PC12 cells and Aβ expression. **(A)** Immunofluorescence staining of PC12 cells in each group. **(B)** Relative expression analysis of Aβ in PC12 cells of each group (n = 3). Data were expressed as mean ± SEM. ^*^P < 0.05, ^**^P < 0.01, and ^***^P < 0.001 vs. Con. ^#^P < 0.05, ^##^P < 0.01, and ^###^P < 0.001 vs. AD.

As shown in Figures 3A,B, Aβ deposition in the AD group was significantly higher than that in the control group (^***^p < 0.001). Compared with the AD group, all tested concentrations of KSG and donepezil significantly reduced Aβ deposition in PC12 cells induced by Aβ_25-35_.

#### 3.4.5 Regulation of different drug concentrations on inflammation and Apoptosis induced by Aβ in PC12 cells

**FIGURE 4:**
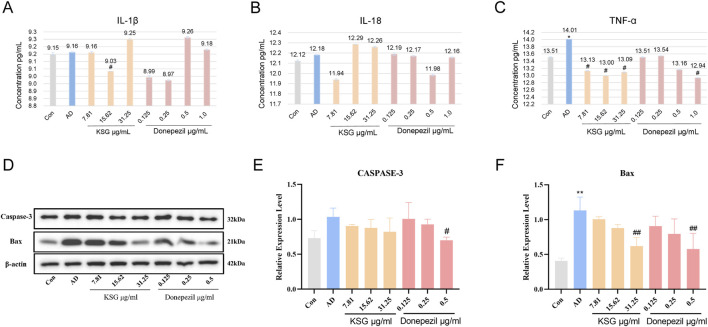
Regulatory effects of KSG on the expression of cytokines and apoptosis-related factors in Aβ-induced PC12 cells. **(A)** Expression of IL-1β (n = 3). **(B)** Expression of IL-18 (n = 3). **(C)** Expression of TNF-α (n = 3). **(D)** Protein expression of apoptosis-related factors in PC12 cells of each group (n = 3). **(E)** Expression of Caspase-3 (n = 3). **(F)** Expression of Bax (n = 3). Data were expressed as mean ± SD. ^*^P < 0.05, ^**^P < 0.01, and ^***^P < 0.001 vs. Con. ^#^P < 0.05, ^##^P < 0.01, and ^###^P < 0.001 vs. AD.

Neuroinflammation is an important mechanism underlying Aβ-induced neuronal death (Zhao et al., 2023). Aβ activates glial and neuronal cells, leading to the production of inflammatory factors such as TNF-α and IL-1β. As shown in Figures 4A–C, TNF-α expression in the AD group was significantly higher than in the control group (^*^p < 0.05). Compared with the AD group, IL-1β expression was significantly decreased at a KSG concentration of 15.62 μg/mL (^#^p < 0.05), and a TNF-α expression was also significantly decreased. At a donepezil concentration of 1.0 μg/mL, TNF-α expression was also significantly decreased (^#^p < 0.05). At donepezil concentrations of 0.125 μg/mL and 0.25 μg/mL, IL-1β expression showed a decreasing trend but without statistical significance (p > 0.05). At a donepezil concentration of 0.5 μg/mL, TNF-α expression also decreased, but the difference was not statistically significant (p > 0.05), possibly due to the large standard deviation (SD) among replicate wells.

The accumulation of hyperphosphorylated tau and Aβ activates apoptotic pathways, in which Bax and Caspase-3 play critical roles. Bax is a critical component in mitochondrial stress-induced apoptosis, while Caspase-3 is the primary effector enzyme that executes apoptosis (Kumari et al., 2023). As shown in Figures 4D–F, compared with the control group, Bax protein expression in the AD group was significantly increased (^**^p < 0.01), while caspase-3 expression was elevated but without reaching statistical significance. Compared with the AD group, donepezil significantly reduced Bax and caspase-3 expression at a concentration of 0.5 μg/mL ^(#^p < 0.05). KSG significantly reduced Bax expression at a concentration of 31.25 μg/mL (^##^p < 0.01).

#### 3.4.6 Effects of different drug concentrations on telomerase activity Telomere length, and POT1 mRNA expression in PC12 cells induced by Aβ

Previous studies have found that upregulating telomerase activity, delaying telomere shortening, and reducing POT1 expression can alleviate Aβ deposition (Li et al., 2021; Qin et al., 2019; Suelves et al., 2023). Therefore, we investigated the effects of various KSG concentrations on Aβ-induced telomerase activity, telomere length, and POT1 mRNA expression in PC12 cells using qPCR.

**FIGURE 5:**
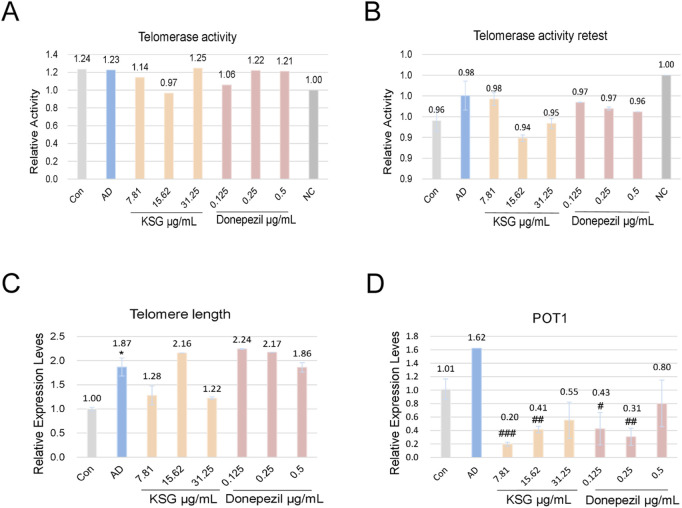
Effects of KSG on telomerase activity, telomere length, and POT1 mRNA expression in Aβ-induced PC12 cells. **(A)** Telomerase activity assay results (n = 3). **(B)** Repeat telomerase activity assay results (n = 3).** (C)** Relative expression of telomere length (n = 3). **(D)** Relative expression of POT1 mRNA (n = 3). Data were expressed as mean ± SD. ^*^P < 0.05, ^**^P < 0.01, and ^***^P < 0.001 vs. Con. ^#^P < 0.05, ^##^P < 0.01, and ^###^P < 0.001 vs. AD.

As shown in Figures 5A,B, telomerase activity was not detected at a KSG concentration of 15.62 μg/mL, while activity was observed in the remaining samples; however, no statistically significant differences were found among the groups. In a repeat assay, no telomerase activity was detected in any group, which was presumed to result from compound degradation or telomerase instability. Taken together, neither experiment showed a significant improvement in telomerase activity by any treatment.

As shown in Figure 5C, compared with the control group, telomere length mRNA expression was significantly increased in the AD group (^*^p < 0.05). Compared with the AD group, KSG at 15.62 μg/mL and donepezil at 0.125 μg/mL and 0.25 μg/mL increased telomere length mRNA expression, but these differences were not statistically significant (p > 0.05). As shown in Figure 5D, POT1 expression in the AD group was higher than that in the control group, though not statistically significant (p > 0.05). Compared with the AD group, POT1 expression decreased across all KSG and donepezil treatment groups, with significant differences observed in all but the highest concentration group (^#^p < 0.05). In conclusion, none of the tested samples significantly enhanced telomere length or regulated POT1 expression. Further investigation is needed to elucidate the underlying mechanisms, particularly in relation to telomerase activity and apoptosis pathways.

### 3.5 Potential mechanisms of KSG analyzed by network pharmacology

#### 3.5.1 Active compounds and potential targets of KSG

**FIGURE 6:**
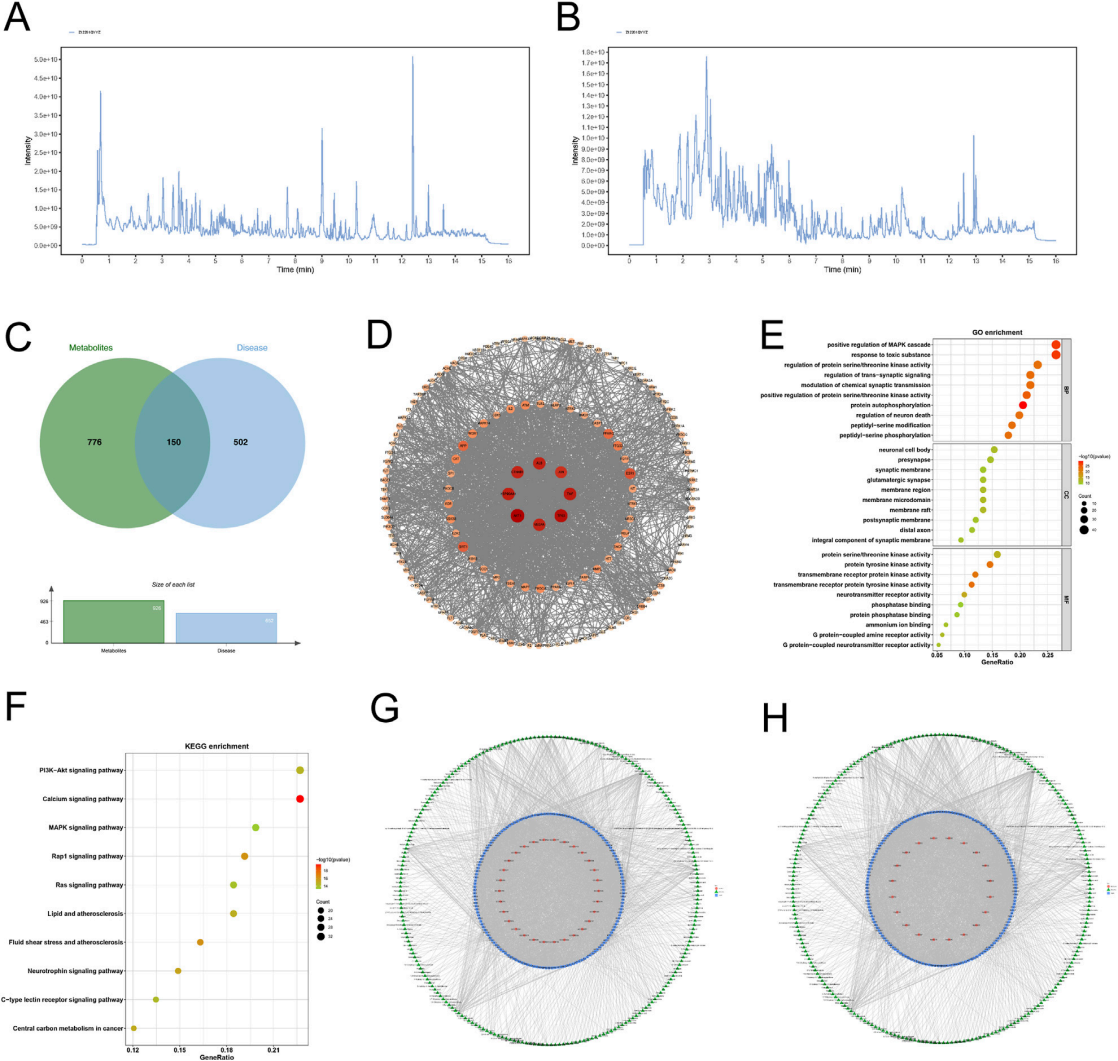
Network pharmacology analysis of KSG components against AD. **(A) **Total ion chromatogram of KSG in positive ion mode. **(B)** Total ion chromatogram of KSG in negative ion mode. **(C)** Venn diagram of intersecting targets between KSG action targets and AD disease targets. **(D)** Protein-protein interaction (PPI) network of core targets. **(E)** Bubble plot of GO enrichment analysis for target proteins stratified. **(F)** Bubble plot of KEGG metabolic pathway enrichment analysis for target proteins. **(G)** Metabolite-target-GO interactive network diagram. **(H)** Metabolite-target-KEGG pathway interactive network diagram.

A total of 445 components were identified through LC-MS/MS analysis, including 316 detected in the positive ion mode and 125 identified in the negative ion mode (Figures 6A,B). For the network pharmacology analysis, 441 substances were identified across both modes, along with 4 compounds (salvianolic acid, ginsenoside Rb1, disinapyl sucrose, echinoside) added via manual curation and nine prioritized compounds (echinoside, 3,6′-disinapyl sucrose, salvianolic acid B, tanshinone IIA, ginsenoside Rg1, ginsenoside Re, Ginsenoside Rb1, catalpol and β-asarum ether). An additional 1,935 compounds were retrieved from the database, resulting in a total of 2,389 substances.

Based on the criteria for oral bioavailability (OB ≥ 30%) and drug-likeness (DL ≥ 0.18), 226 metabolites were selected, yielding 926 associated targets from the database.

#### 3.5.2 Construction of protein interaction network for potential targets of Jiawei Xinxing SAN granules

A total of 652 AD-related therapeutic targets were obtained from the TTD and DisGeNET databases and relevant literature. The intersection of these AD disease targets with KSG-related targets was used to construct a Venn diagram, identifying 150 potential action targets of KSG (Figure 6C). These targets were intersected, and the protein-protein interaction (PPI) relationships of the target proteins were retrieved from the STRING database (*Homo sapiens*) to construct a PPI network (Figure 6D). The resulting network consisted of 149 nodes (targets) and 1,657 edges, with an average node degree value of 0.658. The top ten targets included AKT1 (degree = 86), TP53 (degree = 81), TNF (degree = 77), HSP90AA1 (degree = 76), ALB (degree = 75), CTNNB1 (degree = 74), VEGFA (degree = 74), JUN (degree = 70), ESR1 (degree = 60), and SIRT1 (degree = 56), which may represent the core targets of KSG against AD.

#### 3.5.3 Enrichment analysis

A total of 3,355 GO terms (p < 0.05) were enriched, including 2,939 (87.60%) biological processes (BP), 154 (4.59%) cellular components (CC), and 262 (7.81%) molecular functions (MF). The analysis highlights the top 10 results (Figure 6E). A total of 166 enriched pathways (p < 0.05) were obtained through KEGG Pathway enrichment, and the KEGG Pathway analysis showed the top 10 results (Figure 6F).

#### 3.5.4 Metabolite-target-GO and metabolite-target-KEGG pathway interaction networks

The 226 active ingredients, 150 intersection targets, the top 30 GO terms, and top 20 KEGG pathways were analyzed using R version 3.6.3. The “metabolite-target-GO” and “metabolite-target-KEGG pathway” interaction network were constructed (Figures 6G,H). Key compounds identified based on network degree included Genistein (degree = 50, P < 0.05), Quercetin (degree = 48), and Apigenin (degree = 40). These may be among the primary bioactive substances in the formula. TP53 (degree = 278), AKT1 (degree = 109), PTGS2 (degree = 108) and CNR2 (degree = 104) were identified as potential key targets of this combination. Key pathways included the Calcium signaling pathway (degree = 32), PI3K-Akt signaling pathway (degree = 32), and MAPK signaling pathway (degree = 28), which may be essential pathways for the mechanism of action of this combination. By constructing the “metabolite-target-GO” and “metabolite-target-KEGG pathway” networks, it was demonstrated that different components of Kaixin SAN corresponded to different targets and ultimately exerted their cognitive improvement effects through various signaling pathways, consistent with the multi-component, multi-target, and multi-pathway synergistic characteristics of TCM.

#### 3.5.5 Molecular docking of the key compounds and the targets

**FIGURE 7:**
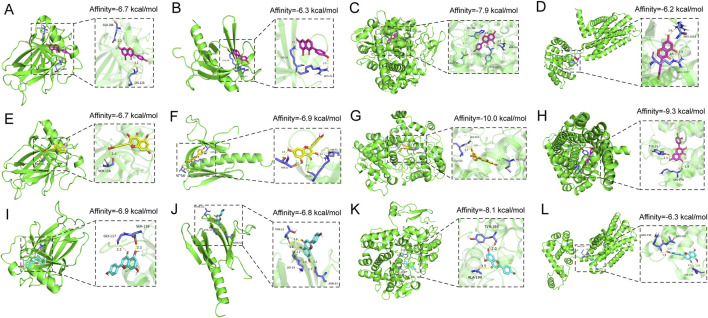
Docking patterns of the key active compounds in KSG and key targets of AD. **(A)** Genistein-TP53, **(B)** Genistein-AKT1, **(C)** Genistein-PTGS2, **(D)** Genistein-CNR2, **(E)** Quercetin-TP53, **(F)** Quercetin-AKT1, **(G)** Quercetin-PTGS2, **(H)** Quercetin- CNR2, **(I)** Apigenin-TP53, **(J)** Apigenin-AKT1, **(K)** Apigenin-PTGS2, **(L)** Apigenin- CNR2.

Molecular docking was conducted between the key components of KSG (Genistein, Quercetin, and Apigenin) and their key targets (TP53, AKT1, PTGS2, and CNR2). The docking results demonstrated that all three active components exhibited favorable binding activity with the targets, with binding affinities of less than −5.0 kcal/mol, meeting the criteria for stable intermolecular binding. The binding energies of each key compound to the selected targets are showed in Figures 7A–L, showing their specific values, reflecting the varying binding capacities of the components to the targets and providing a reference for subsequent mechanistic studies.

#### 3.5.6 Regulatory effects of KSG on key molecules in the PI3K-AKT and MAPK/ERK pathways in the hippocampus of AD model mice

**FIGURE 8:**
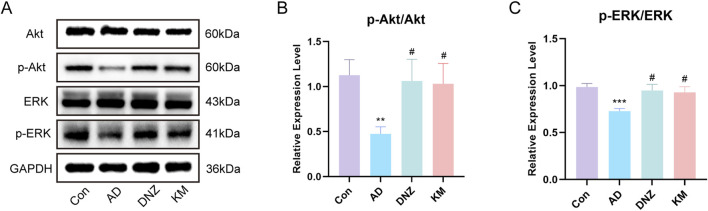
Regulation of PI3K-AKT and MAPK-ERK Pathways by KSG in AD Model Mice. **(A)** Protein expression of Akt, p-Akt, ERK,p-ERK in the hippocampus of mice in each group (n = 3). **(B)** Expression of p-Akt/Akt (n = 3). **(C)** Expression of p-ERK/ERK (n = 3). Data were expressed as mean ± SD. ^*^P < 0.05, ^**^P < 0.01, and ^***^P < 0.001 vs. Con. ^#^P < 0.05, ^##^P < 0.01, and ^###^P < 0.001 vs. AD.

Because of the superior performance of the KH group in the behavioral tests, we validated the core pathways predicted by network pharmacology in the four experimental mouse groups (Con, AD, DNZ, and KH). Western blot analysis revealed the expression and phosphorylation levels of key molecules in the PI3K-AKT and MAPK/ERK pathways in hippocampal tissues (Figure 8A). As shown in Figure 8B, compared to the Con group, the ratio of p-AKT/AKT in the hippocampal tissues of mice in the AD group was significantly decreased (^**^p < 0.01), indicating that the activation of the AKT pathway was inhibited in the scopolamine-induced AD model. In comparison to the AD group, the p-AKT/AKT ratios in the DNZ group and KH group were increased (^#^p < 0.05), indicating that donepezil and high-dose KSG could effectively activate the PI3K-AKT pathway, and that the effect of high-dose KSG was comparable to that of donepezil. Additionally, as depicted in Figure 8C, compared to the Con group, the ratio of p-ERK/ERK in the hippocampal tissues of mice in the AD group significantly decreased (^***^p < 0.001), indicating a reduction in ERK pathway activation in the scopolamine-induced AD model. Compared with the AD group, the p-ERK/ERK ratios in the DNZ group and KH group were significantly elevated (^#^p < 0.05), demonstrating that both donepezil and high-dose KSG could restore the normal activation of the ERK pathway.

## 4 Discussion

KSG is a TCM compound granule formulated developed based on the classic ancient prescription, Kaixin Powder, which has been used for the treatment of amnesia for thousands of years. However, its mechanism of action remains unclear. Through a series of network pharmacology studies, we identified Genistein, Quercetin, and Apigenin as the active compounds in KSG that may contribute to its therapeutic effects. TP53, AKT1, PTGS2, and CNR2 may represent the key targets of KSG. Further molecular docking analyses indicated that the key active components of KSG (genistein, quercetin, and apigenin) exhibited favorable binding activity to the core targets (TP53, AKT1, PTGS2, and CNR2). As a core molecule in the PI3K-AKT pathway, the high-efficiency binding of AKT1 to KSG components may serve as an important molecular basis for the upregulation of p-AKT levels. PTGS2, as a key target in inflammatory regulation, showed stable binding to KSG components, providing a target basis for its role in improving neuroinflammation. Additionally, the involvement of TP53 and CNR2 indicates that KSG may synergistically regulate processes such as cell apoptosis and neuroprotection through multiple targets (Mlost et al., 2018; Thijssen et al., 2021). The Calcium signaling pathway, PI3K-Akt signaling pathway, and MAPK signaling pathway appear to be critical in mediating the therapeutic actions of this combination. In recent years, the role of the Calcium signaling pathway in AD has received increasing attention (Alwiraikat-Flores and Octavio-Aguilar, 2023). Disruption of Ca^2+^ signaling and calcium homeostasis, often resulting from abnormal Ca^2+^ channel function, are believed to play fundamental roles in AD pathogenesis (Popugaeva et al., 2015). Numerous studies have demonstrated that regulation of the Calcium signaling pathway can modulate inflammation and apoptosis, thereby exerting neuroprotective effects and slowing down AD progression (Niu et al., 2024; Xiaojuan et al., 2024). However, due to limitations in experimental conditions, this study was unable to directly observe the regulatory effect of KSG on calcium signaling. Nevertheless, we verified that KSG exerts a mitigating effect on apoptosis and inflammation regulated by calcium signaling. The PI3K/Akt pathway is a key drug target for regulating Aβ deposition and neuronal apoptosis (Seidler and Barrow, 2022). Caruso et al. (2019) demonstrated that an increase in Akt phosphorylation levels can alleviate ROS and reduce Aβ in the lateral ventricle (Caruso et al., 2019). The activation of nicotinic acetylcholine receptors (nAChR) in α7 neurons and Akt signal transduction are associated with the inhibition of Aβ aggregation (Ren et al., 2019). At 28 days after AD induction by Aβ_25-35,_ upregulating Akt expression in the hippocampal CA1 region can improve cognitive impairment (Sun et al., 2020). These are consistent with our findings, which suggest that the PI3K-Akt signaling pathway may be a key pathway through which KSG exerts its effects. Moreover, the activation and inhibition of the MAPK/ERK signaling pathway are known to play vital roles in AD pathogenesis. Aβ levels are regulated by multiple mechanisms. Both synaptic transmission and the activation of certain neurotransmitter receptors can influence Aβ production (Islam et al., 2023). In mice and humans, ERK signaling can be activated by serotonin receptors, thereby increasing the enzymatic activity of α-secretase and reducing Aβ levels (Cirrito et al., 2011; Yuede et al., 2021). Additionally, the activation of AChR allows Ca^2+^ influx, which stimulates the downstream ERK pathway and triggers a series of neuroimmune responses (Kim et al., 2013). Studies have demonstrated that cognitive impairment in AD can be alleviated by regulating the phosphorylation and activation of this pathway (Zhang, Z. et al., 2024). Western blot experiments revealed that in scopolamine-induced AD model mice, KSG could restore the normal levels of ERK/p-ERK by increasing the p-AKT/AKT ratio, which further indicated that KSG may exert anti-AD effects by regulating the PI3K-AKT and MAPK/ERK pathways. Combined with the multi-pathway targeting characteristics predicted by network pharmacology and the molecular docking evidence of multiple active components in KSG with key targets, this demonstrates that the “multi-component, multi-target, multi-pathway” anti-AD effect of KSG. Collectively, these findings suggest that KSG holds promising clinical potential for AD treatment.

To investigate the effects and mechanisms of KSG on learning and memory abilities in AD, we employed the novel object recognition, step-down platform, and Morris water maze tests in a scopolamine-induced AD mouse model. Scopolamine, administered via intraperitoneal injection induces short-term learning and memory impairments, cholinergic dysfunction, oxidative stress, and other pathological features that mimic those seen in AD patients (Kulshreshtha and Piplani, 2016; Xu et al., 2019). Our results demonstrated that KSG could partially alleviate scopolamine-induced cognitive deficits. Specifically, KSG significantly reduced the number of errors and increased latency in the step-down test. In the novel object recognition test, it significantly decreased exploration time for familiar objects while increasing the RI. Furthermore, KSG treatment resulted in a significant increase in both the path-length ratio and time ratio during the probe trial of the Morris water maze test.

Based on these behavioral findings, we further observed that KSG significantly increased Ach levels while decreasing AChE in the hippocampus. This mechanism may be associated with KSG enhancing GSH-Px activity, scavenging free radicals, and mitigating oxidative stress and ROS production. Dysfunction in the cholinergic system and oxidative stress are both critical factors in AD pathogenesis. Cholinergic transmission between cholinergic synapses is widespread in the human central nervous system and plays an essential role in higher brain functions such as memory and learning. The cholinergic system contributes significantly to overall brain homeostasis (Hampel et al., 2018). Ach and AChE are essential components of the system, and their dysfunction is closely linked with cognitive decline in AD (Ju and Tam, 2022). The cholinergic hypothesis is a well-established theory describing severe damage to cholinergic neurons in the basal nucleus of Meynert (NBM), leading to a marked reduction in ChAT activity in the cerebral cortex and hippocampus—a region crucial for learning and memory (Hampel et al., 2018; Ju and Tam, 2022). Additionally, the brain, which is primarily composed of lipids that are highly susceptible to oxidation and exhibits high oxygen consumption, lacks efficient oxidative defense mechanisms, making it particularly vulnerable to oxidative damage (Nasb et al., 2024). The progression of AD is accompanied by the progressive alterations or impairments in brain metabolism, including oxidative stress (Nasb et al., 2024; Plascencia-Villa and Perry, 2021). Oxidative stress not only promotes the production and deposition of Aβ but also directly damages cholinergic neurons. Excessive ROS can attack structures including the cell membrane, mitochondria, and nuclear DNA of cholinergic neurons, ultimately resulting in neuronal death and functional loss (Nasb et al., 2024; Plascencia-Villa and Perry, 2023). Aβ can interact with key components of the cholinergic system, such as AChE and nAChRs. These interactions can inhibit the efficacy of cholinergic transmission and further exacerbate cognitive impairment (Lombardo and Maskos, 2015). GPx, an important antioxidant enzyme, plays a critical role in combating ROS by preventing lipid peroxidation, protecting cell membranes from damage, and delaying the onset of AD (Reichert et al., 2020).

Although the exact pathological mechanism of AD remains unclear, neuronal apoptosis has long been recognized as a key factor in AD pathogenesis. In the brains of clinical AD patients, neuronal apoptosis, accumulation of Aβ plaques, and neurofibrillary tangles (NFTs) are commonly observed (Zhang, Z. et al., 2024). Oxidative stress is considered a key inducer of AD, with excessive production of ROS, leading to the over accumulation of Aβ and inducing neuronal apoptosis (He et al., 2023). The pro-apoptotic protein, Bax, can permeabilize the mitochondrial membrane, disrupt Ca2+ homeostasis, and impair energy metabolism, thereby activating the intrinsic apoptotic pathway (Kumari et al., 2023). Donepezil, an AChE inhibitor, enhances cognitive function in AD patients by inhibiting AChE activity, slowing the breakdown of ACh in the synaptic cleft, and thereby increasing ACh levels. It is currently the most widely approved drug for mild-to-moderate AD treatment (Galimberti and Scarpini, 2016; Oliveira Silva et al., 2024). In this study, donepezil was used as a positive control to investigate the neuroprotective effects of KSG and its underlying mechanisms. Our findings revealed that KSG could alleviate Aβ_25-35_-induced injury in PC12 cells at a specific concentration, whereas donepezil showed no significant effect on Aβ_25-35_-induced reduction in PC12 cell viability. Both KSG and donepezil were capable of attenuating Aβ_25-35_-induced inflammatory responses. Donepezil alleviated Aβ_25-35_-induced apoptosis by downregulating the expression of the pro-apoptotic protein Caspase-3, while KSG most likely exerts its protective effect by upregulating anti-apoptotic protein expression and reducing the expression of pro-apoptotic proteins, thereby mitigating Aβ_25-35_-induced apoptosis. Additionally, both KSG and donepezil significantly reduced Aβ deposition in PC12 cells induced by Aβ_25-35_.

Telomeres are DNA-protein structures that protect chromosome ends, and their length is closely associated with cognitive function. Delaying telomere shortening has been shown to improve cognitive function and prevent AD progression (Broccoli et al., 1997). Telomerase regulates telomere length and improves cognitive performance by extending the 3′end of chromosomes through both enzymatic activity-dependent and -independent mechanisms (Jacobs et al., 2014; Liu et al., 2024; Zvereva et al., 2010). Therefore, telomerase activity is closely linked to AD pathogenesis. The telomeric protective protein POT1, located on telomeric DNA, binds to tripeptidyl peptidase 1 (TPP1) to shield chromosome ends from recognition by cellular repair mechanisms along the 5′to 3′direction (Moye et al., 2015; Sekne et al., 2022). Inhibition of POT1 has been shown to alleviate cellular senescence and AD symptoms while delaying the process of cellular aging (Li et al., 2021). In this study, we found that neither KSG nor donepezil significantly increased telomerase activity, telomere length, or mRNA expression of POT1. Due to the limitations inherent in vitro cell models, further investigation with a larger number of cell samples or animal experiments is warranted to explore the effects of KSG on telomerase activity, telomere length, and POT1 expression.

This study had certain limitations. Firstly, preclinical research using a mouse model of scopolamine intraperitoneal injection is not a perfect representation of clinical practice. The pathological process of clinical AD is complex and progressive, involving multiple pathological processes including β-amyloid deposition, excessive phosphorylation of tau protein, and long-term damage to the central cholinergic system. In contrast, the scopolamine-induced AD model rapidly simulates partial AD-like pathological features such as acute cholinergic deficits and cognitive impairment, but lacks the typical pathological deposition of Aβ and tau proteins, making it difficult to mimic the complex pathological network of clinical AD (Balakrishnan et al., 2023; Jagielska et al., 2025). Therefore, future studies need to accurately simulate the molecular and pathological characteristics of clinical AD through genetic modification based on transgenic animal models, such as APP/PS1, to provide more reliable experimental evidence for the clinical translation of KSG (Long et al., 2024; Toledano et al., 2024). Although network pharmacology suggests that KSG may act through the calcium signaling, PI3K-Akt and MAPK pathways, gene-editing techniques such as CRISPR-Cas9 were not employed to verify the involvement of these specific pathways. Furthermore, this study lacks targeted screening of individual active components of KSG that may target dementia, which could be addressed in future research by investigating individual drug monomers.

## 5 Conclusion

KSG alleviated learning and memory impairments in scopolamine-induced AD mice, potentially by reducing AChE and ROS levels while increasing Ach and GSH-Px levels. These effects contributed to restoring cholinergic function and inhibiting oxidative stress. *In vitro* experiments demonstrated that KSG reduced Aβ_25-35_-induced injury in PC12 cells, cleared Aβ deposition, and suppressed inflammatory responses and apoptosis. The mechanism of action of KSG may be attributed to its key active ingredients, including Genistein, Quercetin, and Apigenin. Its primary molecular targets are TP53, AKT1, PTGS2, and CNR2, which are closely associated with the Calcium signaling pathway, PI3K-Akt signaling pathway, and MAPK signaling pathway. This study explored the feasibility and underlying mechanisms of KSG in treating AD, providing a potential approach for clinical investigations into AD therapy.

## Data Availability

The data presented in the study are deposited in the figshare repository. This data can be found here: https://doi.org/10.6084/m9.figshare.30067714.

## Supplementary material

SUPPLEMENTARY FIGURE 1 Concentration screening of KSG and donepezil, and validation of Aβ modeling concentration. **(A,B)** Concentration-dependent effects on PC12 cell viability for KSG and donepezil (n = 5). **(C‐E)** Effects of Aβ at different concentrations on cell viability after 24/48/72 h treatment (n = 6). **(F‐H)** Validation of cell viability following 24/48/72 h treatment with indicated Aβ concentrations (n = 6). Data were expressed as mean ± SD. *P < 0.05, P < 0.01, and *P < 0.001 vs Con.

SUPPLEMENTARY FIGURE 2 Protective effects of drug concentrations on Aβ‐injured PC12 cell viability. **(A)** Protective effects of various KSG concentrations against Aβ‐induced PC12 cell injury (n = 6). **(B)** Protective effects of different donepezil concentrations on Aβ‐injured PC12 cell viability (n = 6). **(C)** Protective effects of donepezil in an extended concentration range validation assay against Aβ‐induced PC12 injury (n = 6). Data were expressed as mean ± SD. *P < 0.05, P < 0.01, and *P < 0.001 vs 0.
