# Supplementary figures and images for: Kaixin San Jiawei granule improves cognitive function and alleviates neuronal damage in Alzheimer’s disease via multi-component and multi-target mechanisms
Source: Front Pharmacol. 2025 Sep 19;16:1650534. doi: 10.3389/fphar.2025.1650534 (PMC12492016; doi:10.3389/fphar.2025.1650534)

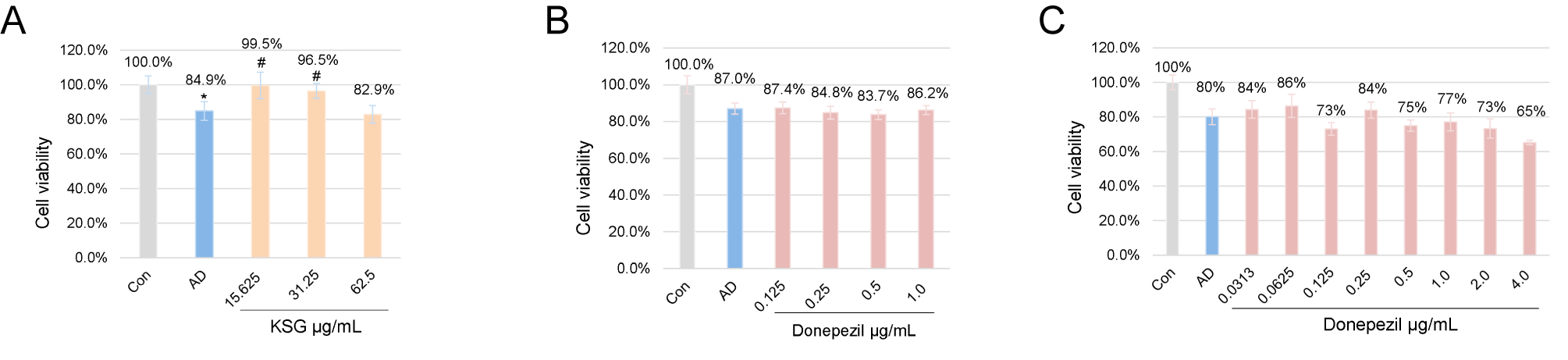

Supplement: Supplementary file 1 [file Image2.tif]

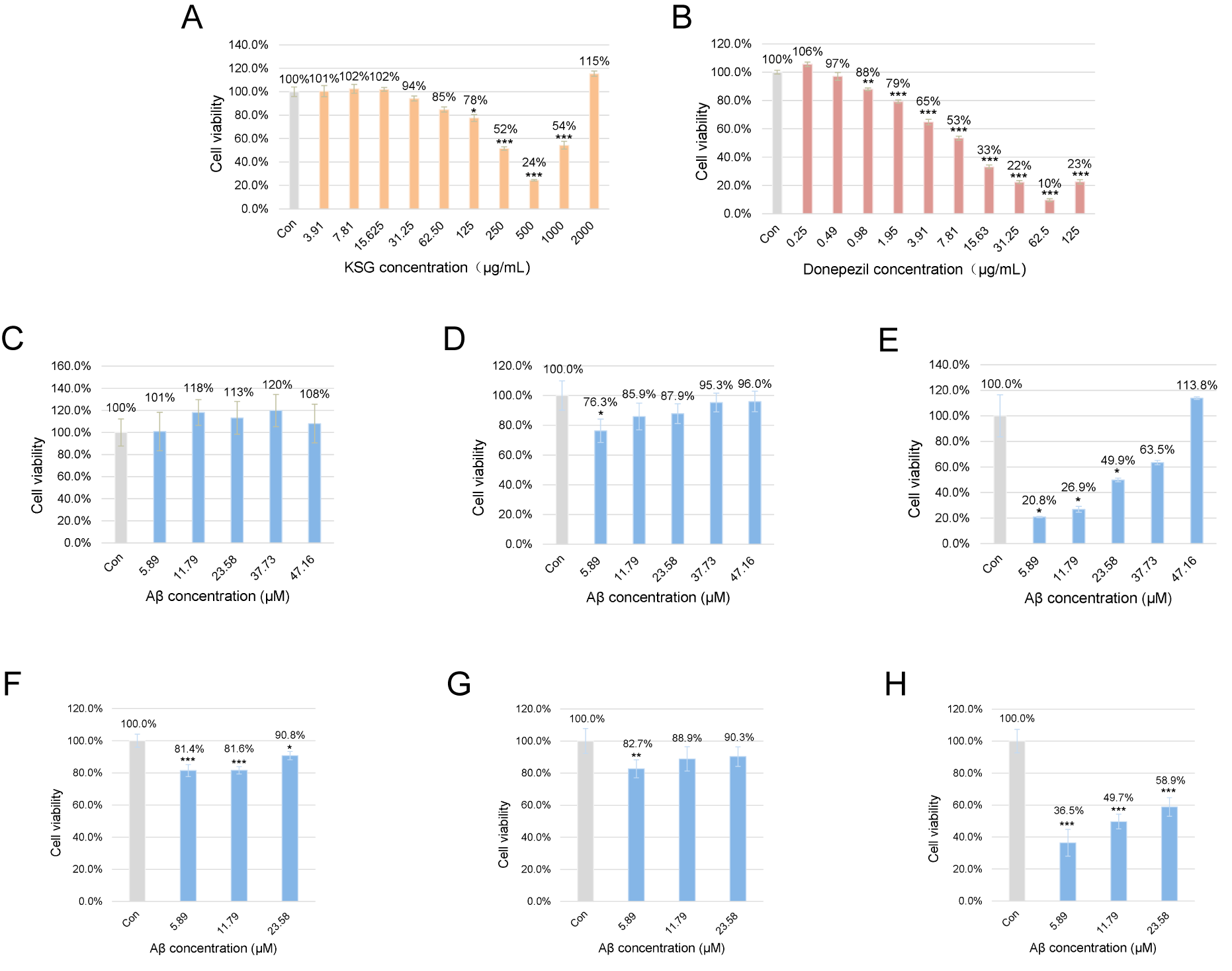

Supplement: Supplementary file 2 [file Image1.tif]
